# Fractional-Order Active Disturbance Rejection Control with Fuzzy Self-Tuning for Precision Stabilized Platform

**DOI:** 10.3390/e24111681

**Published:** 2022-11-17

**Authors:** Jianjian Zhao, Tao Zhao, Nian Liu

**Affiliations:** College of Electrical Engineering, Sichuan University, Chengdu 610065, China

**Keywords:** photoelectric tracking system, fractional-order active disturbance rejection control, fuzzy regulator, disturbance rejection

## Abstract

In this paper, a novel fractional-order active disturbance rejection control with fuzzy self-tuning method (FSFOADRC) is proposed for photoelectric tracking system (PTS). Firstly, aiming at the internal uncertainty of PTS and external disturbance, a fraction-order extended state observer (FOESO) is designed, and the FOESO can transform the plant into a simple form, which greatly simplifies the mathematical model. Secondly, a fuzzy regulator is applied to the proportion–differentiation controller (PD), increasing the flexibility and adaptivity of the controller. In addition, the stability of the whole control system can be guaranteed. Eventually, numerical comparative simulations are implemented to verify the feasibility and superiority of the proposed method. Compared with the integral-order active disturbance rejection control (IOADRC), fractional-order active disturbance rejection control (FOADRC) without the fuzzy regulator and proportion–integration–differentiation (PID) controller, the proposed method performs better with faster response, smaller overshoot, and stronger disturbance suppression capability.

## 1. Introduction

A photoelectric tracking system is an integrated system with a combination of numerous technologies including optics, mechanics, hardware communication, servo control, image processing, and so on, which is widely used in aerospace, ballistic measurement, astronomical observation, laser communication, and quantum communication [1,2,3,4]. With the expansion of the application field of PTS, it is gradually installed on the motion platform. Therefore, higher requirements are proposed for the control algorithms acting on PTS, incorporating more adaptability [5], more intelligence [6], and more maneuverability [7,8]. A PID controller is a classical controller which is broadly applied in most practical control circumstances. However, due to its limitations such as manual adjustment of control parameters, poor anti-interference ability, and inability to solve uncertainty problems, it is not suitable for PTS, which urgently demands high tracking accuracy, strong disturbance suppression capability, and robustness. Hence, it is necessary to develop more intelligent and advanced control strategies with more desired control performance.

After research in recent years, a large number of scholars have attempted to perform progressive control algorithms in PTS. For instance, an improved feedforward control strategy to compensate some time-delay errors was developed for the PTS in [9]. For the problem of disturbances in the working process, an improved control algorithm with the extended state observer based on disturbance frequency adaption was proposed [10]. In addition, a modified smith predictor control scheme was proposed in [11], which enhances the tracking ability of a photoelectric tracking platform. A wide-bandwidth control strategy was employed to improve the closed-loop bandwidth of PTS in [12]. However, these existing studies have the following common problems. Firstly, the controllers are based on an ideal assumption field and the parameters of the controller are fixed, resulting in undesired adaptability, flexibility, and real-time performance. Secondly, these control methods are too complex. Thus, some problems, such as large amount of calculation and excessive number of parameters, lead to excessive and unnecessary cost and are difficult to realize in actual engineering environments. Thirdly, the extant control methods rely too much on accurate mathematical models. When the model parameters change to a certain extent, the control effect becomes unsatisfactory, and the practicability and universality are also poor.

Active disturbance rejection control (ADRC) is an excellent and prevalent control paradigm proposed by Han [13,14]. It is characterized by a strong ability to deal with unknown uncertainties and suppress various external disturbances. The basic idea of ADRC is to create a new state, i.e., “total disturbance”, and uses an extended state observer (ESO) to observe and estimate the total disturbance, so that the feedback control becomes more robust and less dependent on the accurate mathematical model [15]. Then, through compensating the total disturbance, the original complicated and uncertain plant dynamics can be simplified into a cascaded integral form, thus achieving the unification of the model. However, ADRC still has some disadvantages, such as too many parameters, complex tuning process, difficult theoretical analysis, and so on. Therefore, Gao [16,17,18] a applied linear controller and linear extended state observer (LESO) to replace the original nonlinear controller and ESO in ADRC, which greatly simplifies ADRC to linear active disturbance rejection control (LADRC). LADRC possesses the characteristics of simple structure, satisfactory anti-interference ability, and relatively few parameter settings. Accordingly, these properties make LADRC more practical and realistic and greatly promote the in-depth development of ADRC theory. These studies [19,20,21] demonstrate that, through theoretical and experimental proof in frequency domain and time domain, LADRC achieves high performance and strong robustness while simplifying the structure. Although ADRC and LADRC have achieved great success in their respective fields, their inherent integer-order characteristics still limit their application in the occasions with various fractional-order features.

As an extension of integer-order calculus, fractional-order calculus (FOC) plays an extremely significant role in mathematics. It has a history of more than 300 years since it was proposed. Fractional-order control, a new research direction, combines FOC as an essential tool with other control methods [22,23]. Due to the addition of multiple adjustable parameters, the degree of freedom and flexibility of the controller are enhanced, making the control performance more likely to be improved [24], so it has received extensive attention in recent years. For example, scholars [25] proposed a fractional-order internal model controller for fractional-order process model with time delay, which shows that a fractional order controller can obtain more accurate and better results. Ref. [26] proposed a fractional-order nonsingular terminal super-twisting sliding mode control method for a micro-gyroscope with unknown uncertainty, which proves that this control scheme, compared with the integral-order one, can implement better tracking property and estimate the unknown model more accurately. In addition, a radial basis function neural network-based fractional-order control method was proposed for the purpose of improving the control performance for a high-speed train with uncertain model and actuator failures [27]. In addition, the fuzzy regulator, as a intelligent element, which integrates expert experience and effectively uses semantic information to imitate the reasoning process of humans to accurately express fuzzy concepts with mathematical expressions, has a wide application in many fields [28,29,30,31]. Since the parameter settings of the controller seriously affect the control performance of a PTS system, it is possible to boldly try to use the fuzzy regulator to adjust the parameters in the fractional-order control methods adaptively, so as to maintain the high-precision tracking ability and high anti-interference capability in real time.

Accordingly, inspired by the above problems and thoughts, in this paper, a novel fractional-order active disturbance rejection control with fuzzy self-tuning strategy is proposed for a PTS system requiring high-precision control properties. Similar to the traditional IOADRC, FOADRC can simplify the system into a unified form by utilizing the thought of total disturbance as well. Additionally, FOADRC does not rely on accurate mathematical models, and has a strong ability to deal with system uncertainties. In addition, it takes advantage of FOC, which further improves the control performance of the PTS, such as tracking performance and disturbance suppression capacity. Moreover, the flexibility and adaptability of the proposed method can be greatly maintained by the addition of the fuzzy regulator, and the stability analyses of both FOESO and the whole system are discussed. Finally, numerous comparative simulations are carried out to show the superiority and effectiveness of the proposed method.

The major contributions of this research can be summarized as follows:

(1) A novel fractional-order active disturbance rejection control with fuzzy self-tuning is proposed and firstly applied in a photoelectric tracking system, and the bounded-input and bounded-output stability can be proved.
(2) Using the semantic information of the fuzzy regulator and expert knowledge, the controller parameters of FSFOADRC can be adjusted flexibly and in real time, avoiding the disadvantage of manually adjusting the parameters, and greatly increasing the adaptability.
(3) Simulation results show that the proposed control structure can restrain external disturbances and deal with internal uncertainties of the system, and the control performances including transient process and disturbance rejection capability are superior to IOADRC and FOADRC without fuzzy regulator.

The rest of this paper is organized as follows: Section 2 gives a brief description on one of the classical PTSs. Section 3 demonstrates the structure and design process of FSFOADRC. The proof of stability is given in Section 4. Section 5 performs the numerical simulation. Finally, Section 6 presents the conclusion.

## 2. Mathematical Model of Precision Stabilized Platform

**Figure 1:**
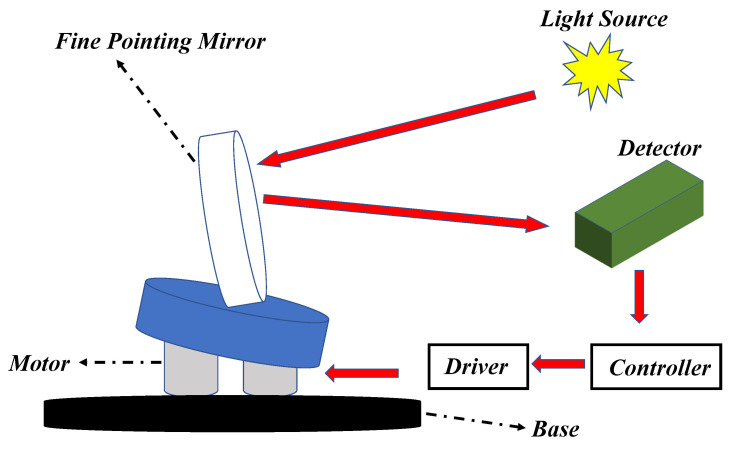
The schematic diagram of a precision stabilized platform.

The precision stabilized platform (PSP) is a typical PTS system and its schematic diagram is shown in Figure 1. The main components of a precision stabilized platform contain a controller, driver modules, a target detector, and a fine pointing mirror. The specific working process is that the fine pointing mirror reflects the beam emitted from light source to the detector, and then the controller produces the rotation angle required by the mirror. After that, through D/A conversion, the output of the controller drives the motor connected to the mirror. The ultimate control objective is to quickly rotate the mirror so that the beam can always be kept at the center of the detector.

In practical engineering, since the frequency characteristics from voltage input to the angle output of the fine pointing mirror can be approximated into a typical resonance element, the model in low and medium frequency can be expressed as a second-order plant, and its transfer function is described in (1) as follows.

(1) $$\begin{array}{c} G_{nom}\left(s\right)=\frac{Y(s)}{U(s)}=\frac{b}{s^{2}+a_{1}s+a_{0}} \end{array}$$

where *U* is the control signal, *Y* is the output, and *b*, $a_{1}$, and $a_{0}$ are nominal parameters.

Through analyzing and fitting the frequency response of the plant, the nominal values of these parameters can be identified. However, the parameters of the model change randomly due to some factors, including self attitude change, base vibration, harsh working environment, and so on, which can be seen as the internal uncertainty of the system.

## 3. Structure of the Proposed Method

**Figure 2:**
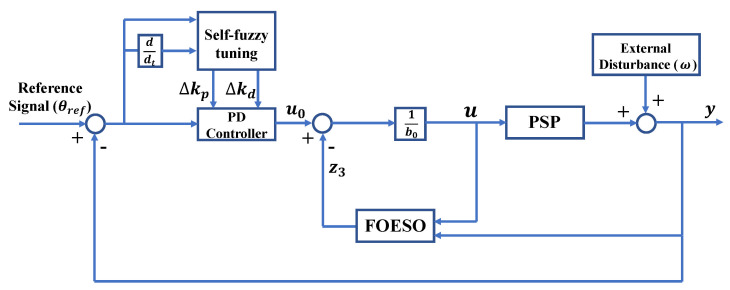
The control structure of FSFOADRC.

The overall control structure of FSFOADRC is displayed in Figure 2. Specifically, FSFOADRC constructs an FOESO, which can both well solve the internal uncertainties of the system and suppress external disturbances. In addition, a fuzzy regulator is applied to adaptively adjust the parameters of the controller, so that the control performances of the system, such as overshoot, settling time, rise time, and so on, are further improved.

### 3.1. Common Definitions of Fractional-Order Derivation

Generally, the common definitions of fractional-order derivation are Riemann–Liouville definition (*R*–*L*), Grünwald–Letnikov definition (*G*–*L*), and Caputo definition. The respective mathematical expressions are as follows.

(1) *R*–*L* definition:
  (2) $${}_{0}^{RL}D_{t}^{\alpha }f\left(t\right)=\frac{1}{\Gamma (n-\alpha )}{\left(\frac{d}{dt}\right)}^{n}\int_{0}^{t}\frac{f(\tau )d\tau }{{\left(t-\tau \right)}^{\alpha -n+1}}$$
(2) *G*–*L* definition:
  (3) $${}_{0}^{GL}D_{t}^{\alpha }f\left(t\right)=\lim_{k\to 0}k^{-\alpha }\sum_{j=0}^{\left[b\right]}{\left(-1\right)}^{j}\left(\begin{array}{c} p\\ j \end{array}\right)f\left(t-jk\right)$$
  In (2) and (3), α is the order and n−1<α<n. When α>0, ${}_{0}D_{t}^{\alpha }f\left(t\right)$ means the α—order differential of integrand f(t). When α<0, ${}_{0}D_{t}^{\alpha }f\left(t\right)$ represents the −α—order integral of integrand f(t), and $\Gamma \left(*\right)$ is the gamma function. *k* is the step size of calculation. $[b]$ is the rounding function of *b*.
(3) Caputo definition:
  (4) $${}_{0}^{C}D_{t}^{\alpha }f\left(t\right)=\frac{1}{\Gamma \left(m-\alpha \right)}\int_{0}^{t}\frac{f^{\left(m\right)}\left(\tau \right)d\tau }{{\left(t-\tau \right)}^{\alpha -m+1}}$$
  where m−1≤α≤m, m∈N, $\Gamma \left(m-\alpha \right)=\int_{0}^{+\infty }{e^{-t}t}^{m-\alpha -1}dt$ is the expression of the gamma function.

In this paper, *R*–*L* fractional-order definition form is adopted.

### 3.2. Design Procedures of FSFOADRC

According to Section 2, the transfer function of the PSP is expressed in (1). Then, transforming (1) into the differential equation

(5) $$\ddot{y}=-a_{1}\dot{y}-a_{0}y+bu+\omega$$

where ω is the external disturbance; then, introducing the fractional-order derivative item

(6) $$y^{\left(2\alpha \right)}=-\ddot{y}-a_{1}\dot{y}-a_{0}y+\omega +y^{\left(2\alpha \right)}+bu=f_{eq}+b_{0}u$$

where $b_{0}\approx b$ and $f_{eq}=-\ddot{y}-a_{1}\dot{y}-a_{0}y+\omega +y^{\left(2\alpha \right)}$ is the total disturbances containing the unknown internal dynamics $-\ddot{y}-a_{1}\dot{y}-a_{0}y$, external disturbance ω, and fractional-order derivative item $y^{\left(2\alpha \right)}$, 0<α<1.

#### 3.2.1. FOESO Design

FOESO introduces the fractional-order derivative operator on the basis of ESO. In addition to observing external disturbances, it can also observe the internal uncertainties of the system, including fractional-order characteristics.

Let $c_{1}=y$, $c_{2}=y^{\left(\alpha \right)}$, $c_{3}=f_{eq}$, $v=f_{eq}^{\left(\alpha \right)}$, and the state-space representation of (6) is

(7) $$\left\{\begin{array}{c} [\begin{array}{c} {c_{1}}^{\left[\alpha \right]}\\ {c_{2}}^{\left[\alpha \right]}\\ {c_{3}}^{\left[\alpha \right]} \end{array}]= [\begin{array}{ccc} 0 & 1 & 0\\ 0 & 0 & 1\\ 0 & 0 & 0 \end{array}] [\begin{array}{c} c_{1}\\ c_{2}\\ c_{3} \end{array}]+ [\begin{array}{c} 0\\ b_{0}\\ 0 \end{array}]u+ [\begin{array}{c} 0\\ 0\\ 1 \end{array}]v\\ y= [\begin{array}{ccc} 1 & 0 & 0 \end{array}] [\begin{array}{c} c_{1}\\ c_{2}\\ c_{3} \end{array}] \end{array}\right.$$

Thus, utilizing the design procedures of the state observer in linear system theory [32], an FOESO can be devised as

(8) $$\left\{\begin{array}{c} [\begin{array}{c} {z_{1}}^{\left[\alpha \right]}\\ {z_{2}}^{\left[\alpha \right]}\\ {z_{3}}^{\left[\alpha \right]} \end{array}]= [\begin{array}{ccc} 0 & 1 & 0\\ 0 & 0 & 1\\ 0 & 0 & 0 \end{array}] [\begin{array}{c} z_{1}\\ z_{2}\\ z_{3} \end{array}]+ [\begin{array}{c} 0\\ b_{0}\\ 0 \end{array}]u+L\left(y-\hat{y}\right)\\ \hat{y}= [\begin{array}{ccc} 1 & 0 & 0 \end{array}] [\begin{array}{c} z_{1}\\ z_{2}\\ z_{3} \end{array}] \end{array}\right.$$

where $z_{i}\;\left(i=1,2,3\right)$ is the state variable of FOESO. $\alpha \left(0<\alpha <1\right)$ is the fractional order of FOESO. $L={ [l_{1},l_{2},l_{3}]}^{T}$ is the observer gain vector, whose value can be designed as $l_{1}=3\omega_{o}$, $l_{2}=3\omega_{o}^{2}$, $l_{3}=\omega_{o}^{3}$ according to the reference [17], and $\omega_{o}$ is the bandwidth of FOESO.

#### 3.2.2. Fuzzy Self-Tuning PD Controller Design

Since the total disturbances $f_{eq}$ can be estimated and compensated via FOESO, and as shown in Figure 2, the control law can be set as

(9) $$u=\frac{u_{0}-z_{3}}{b_{0}}$$

Substituting the above equation into (6), a simple fractional-order integral series form can be obtained, that is

(10) $$y^{\left(2\alpha \right)}\approx u_{0}$$

While the FOESO can estimate the total perturbation exactly, through Laplace transform, (10) can be written as

(11) $$P_{eq}\left(s\right)=\frac{Y(s)}{U_{0}\left(s\right)}=\frac{1}{s^{2\alpha }}$$

According to (10), the original plant is equivalent to a cascaded fractional-order integrator. As the compromise between the stability and the dynamic performance, the phase margin of the plant should preferably be determined as approximately 30° in practical engineering, thus, α in FOESO is selected as 0.75, and a conventional PD controller can keep the system under control and stable. The transfer function of a PD controller is

(12) $$C_{PD}=k_{p}+k_{d}s$$

where $k_{p}$ and $k_{d}$ are proportional and derivative gains, respectively.

However, the PD controller needs manual parameter setting, so the adaptability and flexibility are not satisfactory. In addition, in practical engineering, if the parameters of the model change to some extent, the control performance will be greatly reduced. Hence, for the purpose of achieving desired transient performance and disturbance rejection ability, a fuzzy regulator is designed to intelligently adjust the proportional and derivative gains of the PD controller.

**Figure 3:**
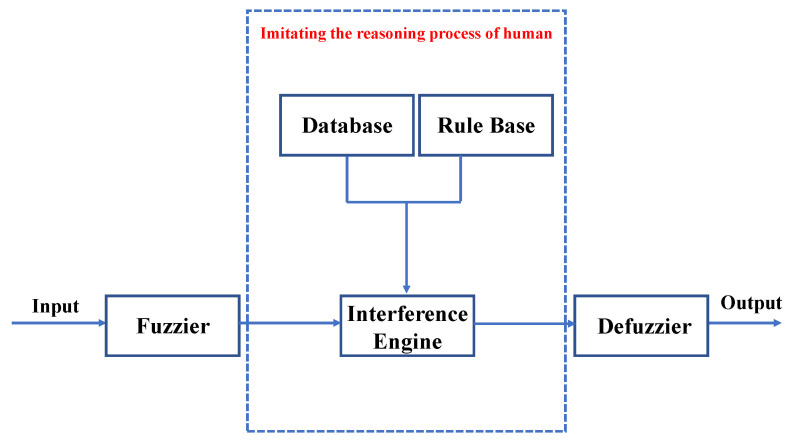
The working diagram of the fuzzy regulator.

The working diagram of the fuzzy regulator is shown in Figure 3. It makes use of expert knowledge, semantic information, etc., through the fuzzy system to adjust the fixed parameters in the system appropriately and adaptively. It can imitate human fuzzy reasoning and decision-making process in behavior. In general, the main components include fuzzier, database, fuzzy inference engine, rule base, defuzzifier, and so on.

According to Figure 2, error and its derivative are the input linguistic variables of the fuzzy regulator, and $\Delta k_{p}$ and $\Delta k_{d}$ are the output variables. We define their fuzzy sets as

(13) $$e,\dot{e},\Delta k_{p},\Delta k_{d}=\left\{NB,NS,ZE,PS,PB\right\}$$

**Figure 4:**
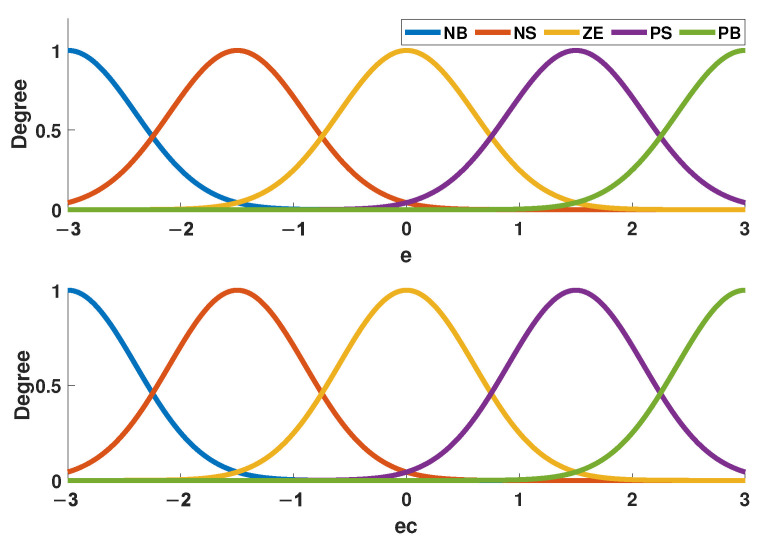
The membership function.

Common membership functions are trimf, Gaussmf, gbellmf, zmf, and so on. In this study, the Gaussian membership is chosen, which is shown in Figure 4, and the range of membership function can be selected flexibly according to different control situations. Moreover, the form of the fuzzy rules is given in (14).

(14) $$\begin{array}{cc} \; & RULE\;i\;:IF\;e\;is\;H_{e}^{i}\;and\;\dot{e}\;is\;H_{\dot{e}}^{j},\\ \; & \;THEN\;\Delta k_{p}\;is\;{ℵ}_{k_{p}}^{o}\;and\;\Delta k_{d}\;is\;{ℵ}_{k_{d}}^{v} \end{array}$$

where $H_{e}^{i}$, $H_{\dot{e}}^{j}$, and ${ℵ}_{k_{p}}^{o}$, ${ℵ}_{k_{d}}^{v}$ (i,j,v,o=1,2,⋯,5) are the fuzzy sets of input variable and output variable, respectively, and the table of fuzzy rules used in Section 5 is shown in Table 1. The basis of determining these fuzzy rules is as follows. For $k_{p}$, the selection of $k_{p}$ depends on the response speed of the system. Increasing $k_{p}$ can improve the response speed and reduce the steady-state error. However, excessive $k_{p}$ will cause large overshoot, or even make the system unstable. Decreasing $k_{p}$ can reduce overshoot and improve stability, but too small $k_{p}$ will slow down the response speed and prolong the settling time. Therefore, in the initial stage of regulation, a relatively large $k_{p}$ should be appropriately taken to improve the response speed, while in the middle stage of regulation, a smaller $k_{p}$ should be taken to make the system have a smaller overshoot and ensure a certain response speed. At the later stage of the adjustment process, $k_{p}$ should be adjusted to a larger value to reduce the static error and improve the control accuracy. For $k_{d}$, the selection of $k_{d}$ has a great influence on the dynamic characteristics. If $k_{d}$ is too large, it will result in a long settling time. If $k_{d}$ is too small, it will lead to an increase in overshoot. According to actual process experience, in order to obtain fast response speed and avoid the possible differential oversaturation in the initial stage, which makes the control effect exceed the allowable range, $k_{d}$ is taken as medium. After that, in order to avoid oscillation near the reference input and take the anti-interference performance of the system into consideration, the value of $k_{d}$ is very important. When the $\dot{e}$ is small, $k_{d}$ is relatively large, and when $\dot{e}$ is large, $k_{d}$ is relatively small. Thus, the fuzzy rules of $\Delta k_{p}$ and $\Delta k_{d}$ can be designed. For instance, if *e* is NB and $\dot{e}$ is NB, then $\Delta k_{p}$ is PB and $\Delta k_{d}$ is PS.

Singleton fuzzifier and product inference engine are applied and the output of the fuzzy system is calculated by the method of center of gravity.

## 4. Stability Analysis

In this section, the stability of FOESO and the stability of the whole system are discussed, respectively.

### 4.1. Stability of FOESO

Let state error be ${e_{i}}^{\left(\alpha \right)}={c_{i}}^{\left(\alpha \right)}-{z_{i}}^{\left(\alpha \right)},i=1,2,3$ and subtract (7) from (8). The state space equation of state error can be written as

(15) $$[\begin{array}{c} {e_{1}}^{\left(\alpha \right)}\\ {e_{2}}^{\left(\alpha \right)}\\ {e_{3}}^{\left(\alpha \right)} \end{array}]= [\begin{array}{ccc} -l_{1} & 1 & 0\\ -l_{2} & 0 & 1\\ -l_{3} & 0 & 0 \end{array}] [\begin{array}{c} e_{1}\\ e_{2}\\ e_{3} \end{array}]+ [\begin{array}{c} 0\\ 0\\ 1 \end{array}]v$$

Hence, the characteristic polynomial of (15) is

(16) $$D\left(s\right)=s^{3\alpha }+l_{1}s^{2\alpha }+l_{2}s^{\alpha }+l_{3}={\left(s^{\alpha }+\omega_{o}\right)}^{3}$$

Let $s^{\alpha }=\omega$, so (16) can be written as

(17) $$D\left(s\right)={\left(\omega +\omega_{o}\right)}^{3}$$

All poles are set at $-\omega_{o}$ and 0<α<1, and the bandwidth $\omega_{o}$ is always positive, so FOESO is bounded-input and bounded-output stable (BIBO).

### 4.2. Stability of the Whole System

**Lemma 1.**  *An ordinary input/output relation (with only integer derivatives) can be written in a polynomial representation*

(18) $$\begin{array}{cc} P\left(\sigma \right)\xi & =Q\left(\sigma \right)u\\ y & =R\left(\sigma \right)\xi \end{array}$$

*where $u\in R^{\overset{-}{m}}$ is the control, $\xi \in R^{\overset{-}{n}}$ is the partial state, and the $y\in R^{\overset{-}{p}}$ is the output; P, Q, and R are polynomial matrices in the variable σ of dimensions $\tilde{n}\times \tilde{n}$, $\tilde{n}\times \tilde{m}$ and $\tilde{p}\times \tilde{n}$, respectively. σ can be seen as the symbol of the usual derivative $s^{\alpha }$, when all initial conditions are zero.*

The proof of lemma above is given in [22].

If the triplet (P,Q,R) of polynomial matrices is minimal, system (18) is bounded-input and bounded-output stable $iff\det\left(P\left(\sigma \right)\right)\neq 0,\forall \sigma ,\;\left|arg\left(\sigma \right)\right|<\frac{\pi \alpha }{2}$.

**Theorem 1.**  *If choosing appropriate parameters α, $k_{p}$, and $k_{d}$ to guarantee $\left|arg\left(\omega_{i}\right)\right|>\lambda \frac{\pi }{2}$, where $\omega_{i}$ is the $i_{th}$ root of equation $\omega^{2p\alpha }+k_{p}+k_{d}\omega^{p}=0$, $\lambda =\frac{1}{p}$, and p is a positive integer, then when $\left.t\to \infty \right.$, the whole closed-loop system is bounded-input and bounded-output stable (BIBO).*

**Proof of Theorem 1.**  The characteristic equation of the whole system is

(19) $$G_{cl}\left(s\right)=1+C_{pd}\left(s\right)P_{eq}\left(s\right)=s^{2\alpha }+k_{p}+k_{d}s=0$$

Notate $\lambda =\frac{1}{p}$, $\omega =s^{\lambda }$, (19) can be written as

(20) $$\omega^{2p\alpha }+k_{p}+k_{d}\omega^{p}=0$$

According to Lemma 1, $\tilde{m}=\tilde{n}=1$, thus

(21) $$\left|arg\left(\omega_{i}\right)\right|>\lambda \frac{\pi }{2}$$

Accordingly, the whole control system is BIBO. □

## 5. Numerical Simulation

In this section, a number of comparative simulations designed in MATLAB are carried out to demonstrate the superior performance controlled by the proposed method. Three relevant and prevalent control methods, i.e., IOADRC, FOADRC, and PID controller, are selected for comparison. The parameters of the nominal model of PSP are determined by some specific methods and can be expressed in (22).

(22) $$G_{nom}\left(s\right)=\frac{Y\left(s\right)}{U\left(s\right)}=\frac{4336.7}{s^{2}+32.67s+1668}$$

where b=4336.7, $a_{1}=32.67$, and $a_{0}=1668$.

However, due to the influence of interference and special working environment, the parameters *b*, $a_{1}$, and $a_{0}$ often change in a random range during the working process, that is, some uncertainties Δb, $\Delta a_{1}$, and $\Delta a_{0}$ are generated, which can be considered as internal uncertainty.

In general, in practical engineering situations, in order to ensure the stability of the system, the phase margin of the system should be equal to or greater than $30^{\circ }$, and owing to the particularity of PSP, its crossing frequency cannot be set very large. Therefore, based on the engineering experience, the simulations in this paper set the phase margin and crossing frequency of the system to be $35^{\circ }$ and 10 rad/s, respectively. According to these frequency-domain indices, the parameters of the PD controller can be designed and determined. Thus, the controllers of IOADRC and FOADRC are shown in (23) and (24), respectively, and the nominal controller of the proposed method is the same as that of FOADRC.

(23) $$G_{IOADRC}\left(s\right)=167.2+13.46s$$

(24) $$G_{FOADRC}\left(s\right)=254.8+15.79s$$

**Figure 5:**
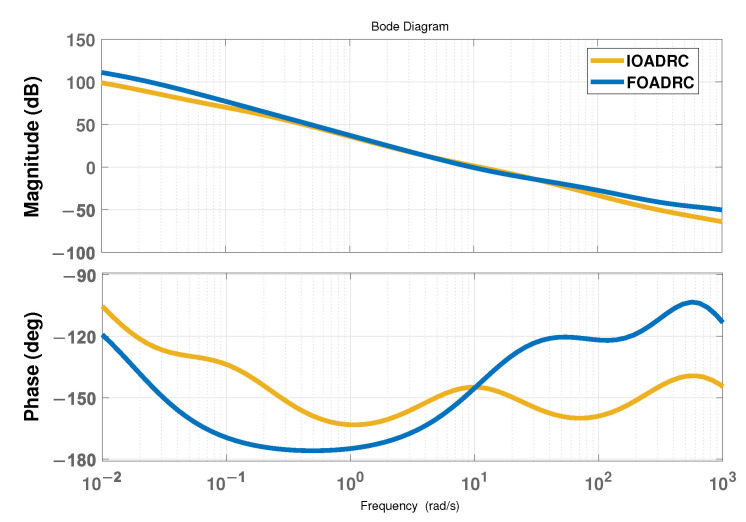
The Bode plot.

The Bode plot of the systems controlled by IOADRC and FOADRC, which meets the predetermined frequency indices, is shown in Figure 5. According to Figure 5, the low-frequency band of FOADRC is higher than that of IOADRC, indicating that FOADRC has better steady-state performance. In addition, the slope of the middle-frequency band of FOADRC is slightly smaller than that of IOADRC, representing that the transient performance of FOADRC is better than that of IOADRC. However, in the high-frequency band, FOADRC is slightly higher, implying that the effect of suppressing high-frequency noise is slightly worse, but the impact in actual engineering is acceptable.

### 5.1. Comparison with IOADRC

**Figure 6:**
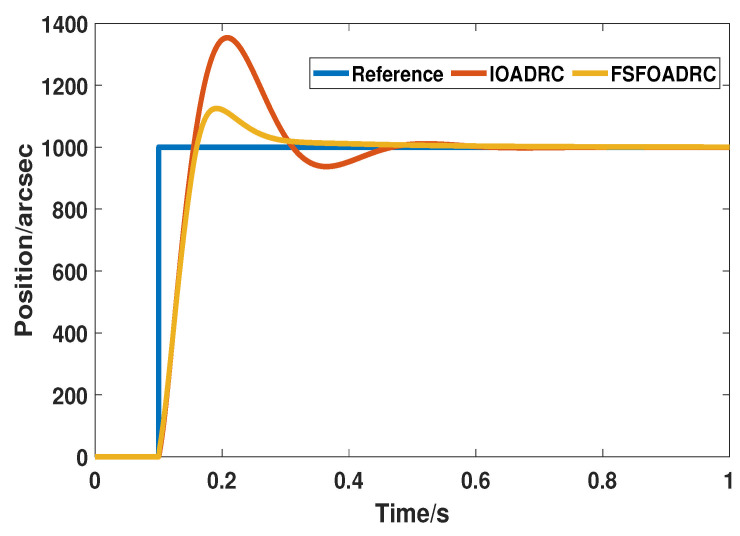
The responses of IOADRC and the proposed method.

Firstly, IOADRC is chosen for comparison. A step signal whose value is 1000 is set as the reference input signal, and the control responses of IOADRC and the proposed method are shown in Figure 6. In addition, the transient performance indices controlled by these two methods, including overshoot, settling time, and peak time, are listed in Table 2.

As shown in Figure 6 and Table 2, qualitatively, the response of the proposed method is more desired than that of IOADRC. Specifically and quantitatively, the overshoots of the proposed method and IOADRC are 12.4% and 35.4%, respectively. Compared with the counterpart, the overshoot of the proposed method has dropped by about 23%, which is a great declination. In addition, the response of IOADRC crosses the reference signal four times during the period of 0.1 s to 0.6 s, but the response of the proposed method descends immediately and tracks the reference signal smoothly after reaching the top, without unnecessary vibration, indicating that the proposed method has a smoother dynamic process and smaller oscillation range and number than IOADRC. Moreover, the settling time and peak time of the proposed method are 0.252 s and 0.1895 s, respectively, which are faster than the 0.297 s and 0.219 s of IOADRC. The shorter settling time and peak time represents that the proposed method is more sensitive to variable signals and can track the reference input signal more rapidly. In addition, both methods can eventually track the reference input signal with minimal steady-state error. Accordingly, in terms of the transient performance, the proposed method, compared with IOADRC, has smaller overshoot, faster response speed, and smaller oscillation times.

**Figure 7:**
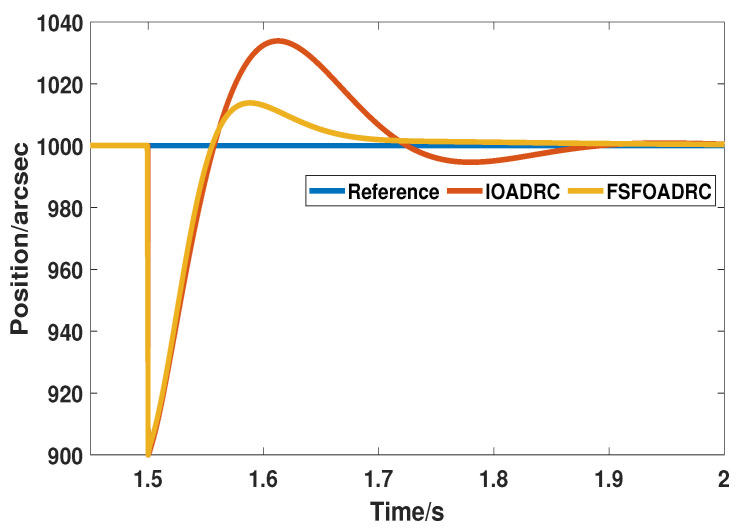
The responses of IOADRC and the proposed method under the step disturbance.

In addition to transient performance, disturbance rejection ability is also an exceedingly crucial indicator for PSP. Since the step disturbance is a common, nonlinear, and abrupt disturbance, and while it can be suppressed, other perturbations, such as slope perturbation, acceleration perturbation, and so on, can also easily be restrained. Thus, on the basis of the above simulations, a step disturbance whose value is 100 is added at 1.5 s as an external disturbance to validate the superior anti-disturbance capacity of the proposed method. The responses controlled by IOADRC and the proposed method are displayed in Figure 7.

As shown in Figure 7, after the system is disturbed, the proposed method can quickly recover to the steady state in about 1.7 s without crossing the reference input again. On the contrary, IOADRC crosses the reference signal and slowly returns to the stable state at about 1.85 s. In addition, the oscillation range of the proposed method is about 900 to 1013, while that of IOADRC is 900 to 1037, indicating that the proposed method can quickly respond to external adverse factors and suppress interference. Therefore, in the case of step disturbance, the proposed method can respond faster and more smoothly on IOADRC, which proves that FSFOADRC has stronger anti-interference ability than IOADRC.

To sum up, when applying the fractional-order calculus, the control effect containing the transient performance and disturbance suppression capability is superior to that using integral calculus.

### 5.2. Comparison with FOADRC

In this subsection, FOADRC is performed for comparison. In order to ensure the fairness and effectiveness of the simulation, the initial parameters of the controller in the proposed method are the same as those of FOADRC.

**Figure 8:**
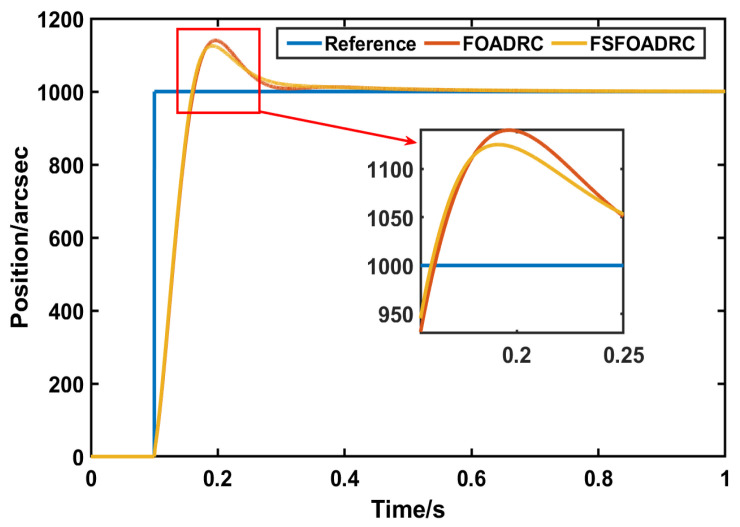
The responses of FOADRC and the proposed method.

**Figure 9:**
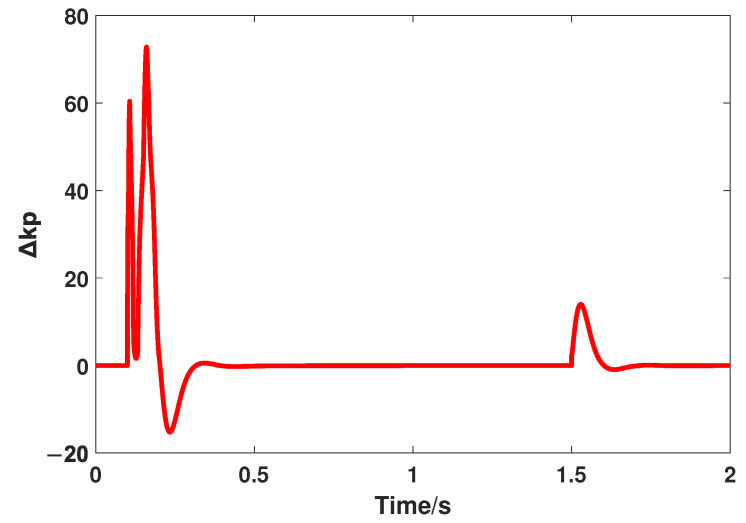
The variation of $\Delta k_{p}$.

**Figure 10:**
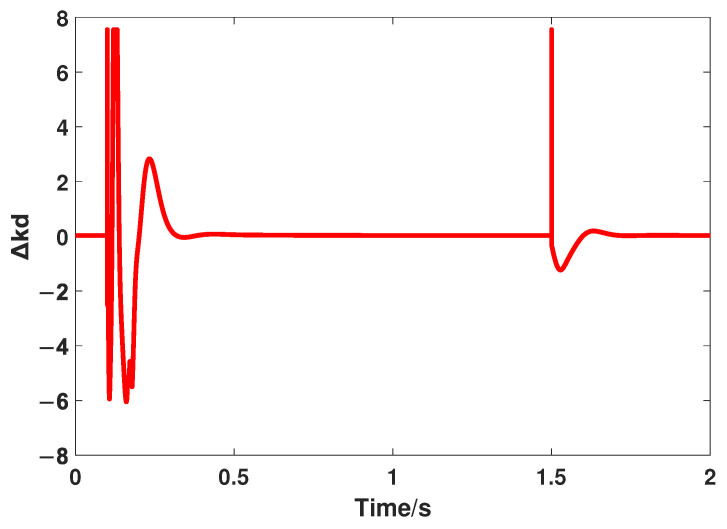
The variation of $\Delta k_{d}$.

Similar to the above part, the transient performance is the first comparative indicator, and the reference input signal is the same, that is, a step signal whose value is 1000. The responses controlled by FOADRC and the proposed method are shown in Figure 8, and the variation of $\Delta k_{p}$ and $\Delta k_{d}$ are shown in Figure 9 and Figure 10, respectively. In addition, the quantitative analysis is listed in Table 3.

According to Figure 8, it is noted that the transient performance of FOADRC and the proposed method is close. However, when the fuzzy regulator is added to adjust the parameters adaptively, the overshoot of the proposed method response is reduced by 1.6% under the condition that the settling time is basically unchanged. In addition, at the beginning of the response, the proposed method is faster, which can be seen from the peak time (the peak time of the proposed method is 0.1895 s, while the peak time of FOADRC is 0.1965 s, a decrease of approximately 0.07 s). Accordingly, the addition of the fuzzy regulator can improve the transient performance to some degree.

**Figure 11:**
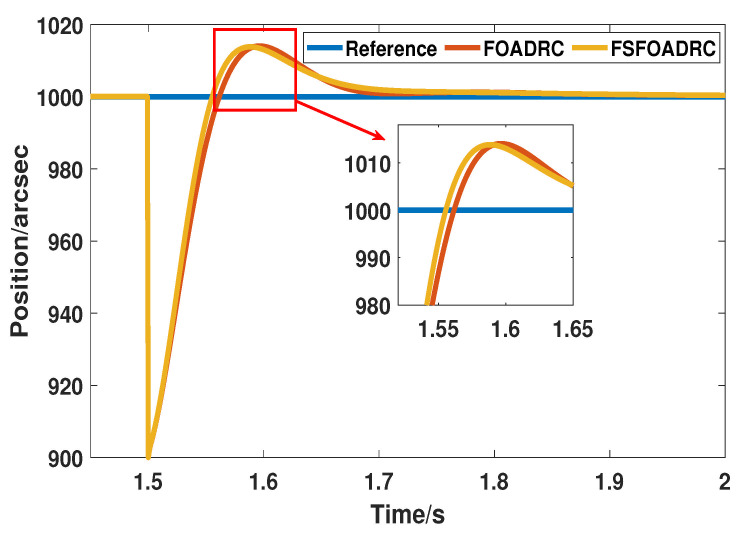
The responses of FOADRC and the proposed method under the step disturbance.

Moreover, the disturbance rejection ability also should be compared. The simulation condition is the same as well, i.e., a step disturbance as the external whose value is 100 perturbation is added at 1.5 s, and the responses are shown in Figure 11.

As shown in Figure 11, it is apparent that the proposed method can return to the reference input signal while the system suffers from the impact of external disturbance, since the response of the proposed method is in front of that of FOADRC. Additionally, compared with FOADRC, the maximum oscillation value of the proposed method is decreased by about 5%. The certain improvement of both the transient performance and disturbance rejection capacity indicates that the fuzzy regulator exactly exerts a positive influence on the whole system.

### 5.3. Comparison with PID Controller

**Figure 12:**
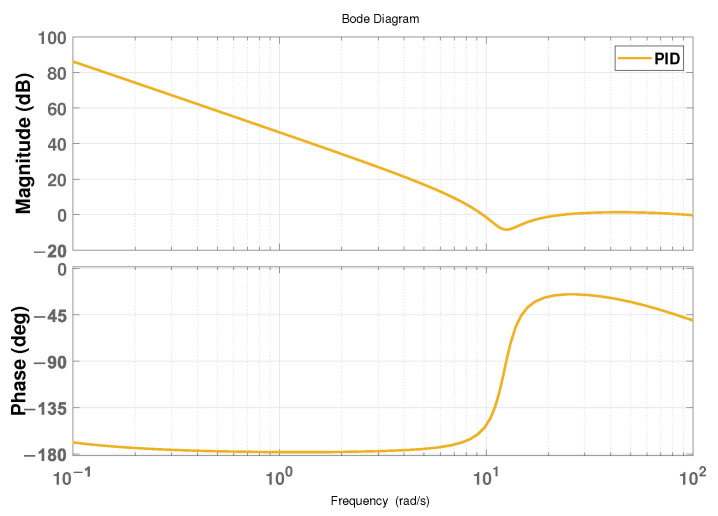
The Bode plot of the system controlled by the PID controller.

In addition to comparing with IOADRC and FOADRC, the PID controller, as one of the most widely used and classic controllers in practical engineering, is implemented as another comparison method to reflect the advantages of the proposed method in this paper, and the simulation conditions are the same as well. For the fairness and effectiveness of the simulation, the frequency indices of the system controlled by the PID controller should be the same, that is, the phase margin and the crossing frequency are approximately $35^{\circ }$ and 10 rad/s, respectively. The Bode plot is shown in Figure 12. In addition, according to the frequency-domain performance properties, the PID controller can be calculated and expressed as

(25) $$G_{PID}\left(s\right)=80.45+\frac{4.87}{s}+0.51s$$

**Figure 13:**
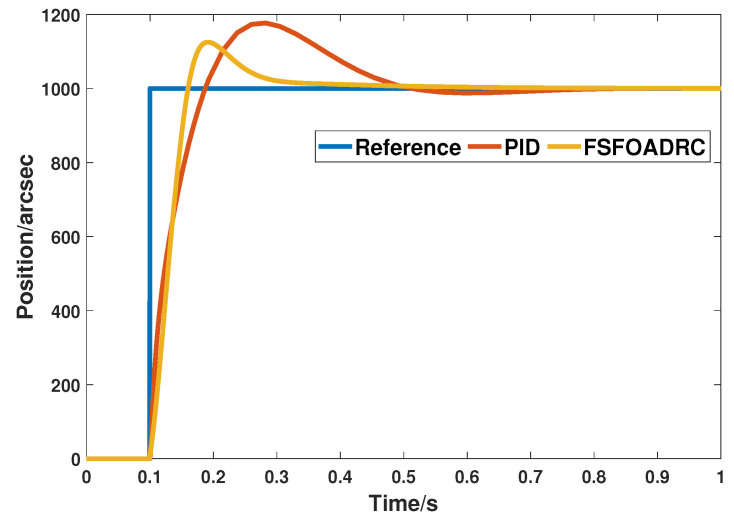
The transient responses of the proposed method and PID controller.

The comparison of transient response is shown in Figure 13, and the corresponding transient control performances are listed in Table 4.

From Figure 13 and Table 4, it is obvious that the overshoot of the proposed method, i.e., 12.4%, is much smaller than that of the PID controller, i.e., 17.7%. In addition, the rapidity of the system response controlled by the proposed method is also better than that of the PID controller, in which the settling time and peak time of PID controller are 0.427 s and 0.282 s, respectively. It also demonstrates that compared with the fixed controller parameters, adding fuzzy self-tuning can make the controller more flexible and adaptive and changes the parameters rapidly according to the tracking input to achieve the aim of obtaining excellent transient performance.

**Figure 14:**
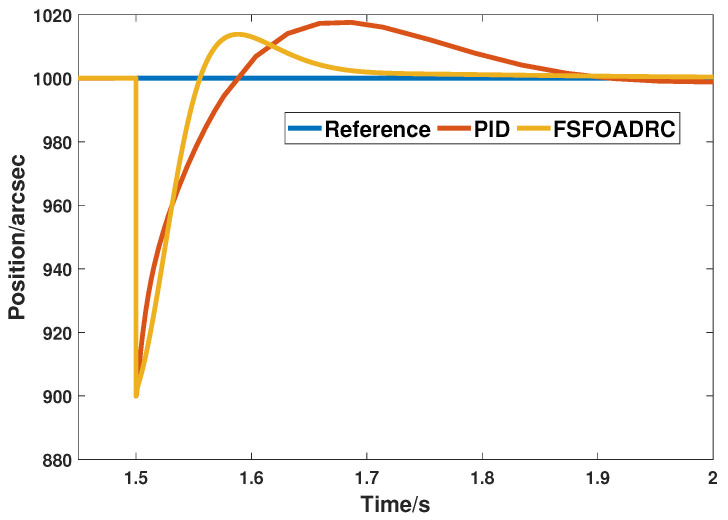
The responses of PID and the proposed method under the step disturbance.

Moreover, the simulation of comparing disturbance suppression ability between the proposed method and PID controller is performed. The result is displayed in Figure 14.

It can be seen from Figure 14 that the response controlled by the proposed method can reach the steady state at approximately 1.7 s, while that of the PID requires 0.2 s more to reach it under the condition of disturbance, and the vibration range of the response controlled by the proposed method when restraining disturbance, i.e., approximately from 900 to 1015, is apparently smaller than that of the PID controller, i.e., approximately from 900 to 1019. This result indicates that the anti-disturbance capability of the PID controller is indeed inferior to that of the proposed method which applies fuzzy self-tuning and FOADRC.

Finally, the integral time absolute error index (ITAE), which can comprehensively reflect the overall control effect of the system, of each method is listed in Table 5. Obviously, the proposed method has the smallest ITAE value.

Overall, through a large number of previous simulations and data, it is verified that the proposed method can achieve a more rapid and smooth transient process and possesses stronger anti-disturbance property compared with IOADRC, FOADRC, and the PID controller.

## 6. Conclusions

Aiming at the PSP with internal uncertainty and external disturbance, an FOADRC control method based on fuzzy self-tuning is proposed in this paper. First of all, the FOESO can not only observe the internal state and total disturbance of the system, but also simplifies the original complex plant, which makes the whole system simpler and the controller easier to design. Secondly, using semantic information and prior knowledge, the fuzzy regulator simulates the human reasoning process and further improves the adaptivity and flexibility. Finally, numerous comparative simulations are carried out to validate the feasibility and effectiveness of the proposed method, namely, the transient performances including overshoot, settling time, and so on, and the anti-interference capacity of the proposed method is superior to IOADRC, FOADRC, and the classic PID controller.

## Figures and Tables

**Table 1:** The fuzzy rules.

$\dot{e}$	NB	NS	ZE	PS	PB
*e*					
NB	PB PS	PB ZE	PB ZE	PS ZE	ZE PS
NS	PB NB	PS NB	PS NS	ZE ZE	NB PS
ZE	PB NB	PS NB	ZE NS	NS ZE	NB PS
PS	PS NB	ZE NS	NS NS	NS ZE	NB PS
PB	ZE PS	NS ZE	NS ZE	NB ZE	NB PS

**Table 2:** Comparison of the transient performance between the proposed method and IOADRC.

Method	Overshoot (%)	Settling Time (Δ=±5%, s)	Peak Time (s)
FSFOADRC	12.4	0.252	0.1895
IOADRC	35.4	0.297	0.219

**Table 3:** Comparison of the transient performance between the proposed method and FOADRC.

Method	Overshoot (%)	Settling Time (Δ=±5%, s)	Peak Time (s)
FSFOADRC	12.4	0.252	0.1895
FOADRC	14	0.255	0.1965

**Table 4:** Comparison of the transient performance between the proposed method and PID.

Method	Overshoot (%)	Settling Time (Δ=±5%, s)	Peak Time (s)
FSFOADRC	12.4	0.252	0.1895
PID	17.7	0.427	0.282

**Table 5:** ITAE comparison of several methods.

	IOADRC	FOADRC	PID	FSFOADRC
$J_{ITAE}$	4788.15	3943.56	4224.49	3870.32

## Data Availability

Not applicable.

## References

[1] Wang J.Y., Yang B., Liao S.K., Zhang L., Shen Q., Hu X.F., Wu J.C., Yang S.J., Jiang H., Tang Y.L. (2013). Direct and full-scale experimental verifications towards ground—satellite quantum key distribution. Nat. Photon.

[2] Yue F., Li X. (2018). Robust adaptive integral backstepping control for opto-electronic tracking system based on modified LuGre friction model. ISA Trans..

[3] Zhao T., Tong W., Mao Y. (2022). Hybrid Non-singleton Fuzzy Strong Tracking Kalman Filtering for High Precision Photoelectric Tracking System. IEEE Trans. Ind. Inform..

[4] Zhang X., Li H. (2021). Research on target detection probability calculation method of photoelectric detection system. Optik.

[5] Lei X., Zou Y., Dong F. (2015). A composite control method based on the adaptive RBFNN feedback control and the ESO for two-axis inertially stabilized platforms. ISA Trans..

[6] Tang T., Niu S., Chen X., Qi B. (2019). Disturbance Observer-Based Control of Tip-Tilt Mirror for Mitigating Telescope Vibrations. IEEE Trans. Instrum. Meas..

[7] Rabinovich W.S., Moore C.I., Mahon R., Goetz P.G., Burris H.R., Ferraro M.S., Murphy J.L., Thomas L.M., Gilbreath G.C., Vilcheck M. (2015). Free-space optical communications research and demonstrations at the U.S. Naval Research Laboratory. Appl. Opt..

[8] Yan W., Liu Y., Lan Q., Zhang T., Tu H. (2022). Trajectory planning and low-chattering fixed-time nonsingular terminal sliding mode control for a dual-arm free-floating space robot. Robotica.

[9] Tang T., Ge R., Ma J., Fu C. (2010). Compensating for some errors related to time delay in a charge-coupled-device-based fast steering mirror control system using a feedforward loop. Opt. Eng..

[10] Wang L., Liu X., Wang C. (2019). Disturbance frequency adaptive control for photo-electric stabilized platform based on improving extended state observation. Optik.

[11] Ren W., Luo Y., He Q., Zhou X., Deng C., Mao Y., Ren G. (2018). Stabilization Control of Electro-Optical Tracking System With Fiber-Optic Gyroscope Based on Modified Smith Predictor Control Scheme. IEEE Sens. J..

[12] Xiao R., Xu M., Shao S., Tian Z. (2019). Design and wide-bandwidth control of large aperture fast steering mirror with integrated-sensing unit. Mech. Syst. Signal Process..

[13] Han J. (1998). Auto-disturbances-rejection Controller and Its Applications. Control. Decis..

[14] Han J.Q. (2002). From PID to active disturbance rejection control. Control Eng. Highl. Ranch- Cahners Then Reed Bus. Inf..

[15] Han J. (1995). The “Extended State Observer” of a Class of Uncertain Systems. Control. Decis..

[16] Gao Z. Active disturbance rejection control: A paradigm shift in feedback control system design. Proceedings of the American Control Conference.

[17] Gao Z. Scaling and bandwidth-parameterization based controller tuning. Proceedings of the 2003 American Control Conference.

[18] Gang T., Gao Z. Frequency Response Analysis of Active Disturbance Rejection Based Control System. Proceedings of the 2007 IEEE International Conference on Control Applications.

[19] Zheng Q., Gao L.Q., Gao Z. (2012). On Validation of Extended State Observer Through Analysis and Experimentation. J. Dyn. Syst. Meas. Control.

[20] Wang Y., Tao L., Wang P., Ma X., Cheng P., Zhao D. (2022). Improved Linear ADRC for Hybrid Energy Storage Microgrid Output-Side Converter. IEEE Trans. Ind. Electron..

[21] Li P., Wang L., Zhong B., Zhang M. (2022). Linear Active Disturbance Rejection Control for Two-Mass Systems Via Singular Perturbation Approach. IEEE Trans. Ind. Inform..

[22] Matignon D. (1996). Stability results for fractional differential equations with applications to control processing. Comput. Eng. Syst. Appl..

[23] Matignon D. (1997). Some Results On Controllability And Observability Of Finite-Dimensional Fractional Differential Systems. Comput. Eng. Syst. Appl..

[24] Podlubny I. (1999). Fractional-order systems and PI/sup /spl lambda//D/sup /spl mu//-controllers. IEEE Trans. Autom. Control.

[25] Arya P.P., Chakrabarty S. (2020). A Robust Internal Model-Based Fractional Order Controller for Fractional Order Plus Time Delay Processes. IEEE Control Syst. Lett..

[26] Fei J., Feng Z. (2021). Fractional-Order Finite-Time Super-Twisting Sliding Mode Control of Micro Gyroscope Based on Double-Loop Fuzzy Neural Network. IEEE Trans. Syst. Man, Cybern. Syst..

[27] Ge M., Song Q., Hu X., Zhang H. (2020). RBFNN-Based Fractional-Order Control of High-Speed Train With Uncertain Model and Actuator Failures. IEEE Trans. Intell. Transp. Syst..

[28] (2022). Coordinated optimization on energy capture and torque fluctuation of wind turbines via variable weight NMPC with fuzzy regulator. Appl. Energy.

[29] Zhao T., Cao H., Dian S. (2022). A Self-Organized Method for a Hierarchical Fuzzy Logic System based on a Fuzzy Autoencoder. IEEE Trans. Fuzzy Syst..

[30] Zhao T., Chen C., Cao H. (2022). Evolutionary self-organizing fuzzy system using fuzzy-classification-based social learning particle swarm optimization. Inf. Sci..

[31] Zhao T., Chen C., Cao H., Dian S., Xie X. (2022). Multiobjective Optimization Design of Interpretable Evolutionary Fuzzy Systems With Type Self-Organizing Learning of Fuzzy Sets. IEEE Trans. Fuzzy Syst..

[32] Wei Y., Ying L., Pi Y.G. (2013). Fractional order modeling and control for permanent magnet synchronous motor velocity servo system. Mechatronics.

